# Taxonomical Investigation, Chemical Composition, Traditional Use in Medicine, and Pharmacological Activities of *Boswellia sacra* Flueck

**DOI:** 10.1155/2022/8779676

**Published:** 2022-02-18

**Authors:** Mansour Miran, Keyvan Amirshahrokhi, Yousef Ajanii, Reza Zadali, Maxwell W. Rutter, Ayesheh Enayati, Farahnaz Movahedzadeh

**Affiliations:** ^1^Department of Pharmacognosy, School of Pharmacy, Ardabil University of Medical Sciences, Ardabil, Iran; ^2^Department of Pharmacology, School of Pharmacy, Ardabil University of Medical Science, Ardabil, Iran; ^3^Botany Division, Research Institute of Forests and Rangelands, Agricultural Research Education and Extension Organization (AREEO), Tehran, Iran; ^4^Department of Pharmacognosy, Faculty of Pharmacy, Islamic Azad University-Damghan Branch, Iran; ^5^Hollingbery & Son, Inc, Yakima, WA 98902, USA; ^6^Ischemic Disorders Research Center, Golestan University of Medical Sciences, Gorgan, Iran; ^7^Institute for Tuberculosis Research, University of Illinois at Chicago, Chicago, IL, USA; ^8^Department of Pharmaceutical Sciences, University of Illinois at Chicago, Chicago, IL, USA

## Abstract

Aromatic oleo-gum-resin secreted from *B. sacra*, reputed as frankincense, is widely used in traditional medicine to treat Alzheimer's disease, gastric disorders, hepatic disorders, etc. Frankincense is also used in the cosmetic, perfume, and beverage and food industries. Frankincense is a rich resource for bioactive compounds, especially boswellic acids and derivatives. Although several reports have described frankincense's constituents and pharmacological activities, there is no comprehensive study that covers the valuable information on this species. Therefore, the current review will focus on the phytochemistry, traditional uses, and pharmacological activities of *B. sacra*.

## 1. Introduction

**Figure 1:**
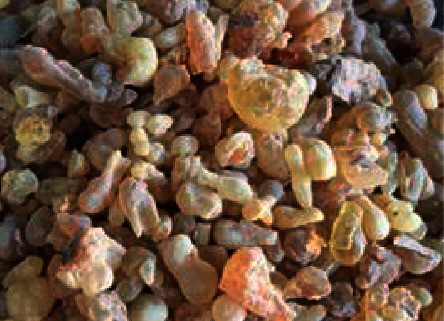
A photograph of frankincense gum resin taken from https://nagaadgums.com.

*Boswellia sacra* Flueck is a perennial plant belonging to the family Burseraceae. Aromatic oleo-gum-resin extracted from *B. sacra*, known as frankincense or olibanum (Figure 1), is used as a home remedy, especially in Middle East countries [1]. Frankincense is harvested by making shallow incisions into the tree trunk [2]. Frankincense is used in many industries such as cosmetic, pharmaceutical, beverage, food, detergents, and perfume industries [3, 4]. The oleo-gum resin of *B. sacra* has many uses for the human body, including analgesic, hepatoprotective, anticoagulant, antioxidant, tumor-suppressive, anti-inflammatory, cardioprotective, Alzheimer's disease, gastric, hepatic, and skin disorders [5, 6]. *B. sacra* is an important source of bioactive compounds, including terpenoids which have a wide range of biological activities [6]. The most important compounds found in resin from *Boswellia sacra* are boswellic acids and their derivatives, which are responsible for a number of medicinal properties belonging to the plant.

Accordingly, this review introduces *B. sacra* as a valuable herbal source in different industries such as pharmacy and food.

## 2. Genus *Boswellia* Roxb. Ex. Colebr

The genus *Boswellia* belongs to the family Burseraceae. It comprises 20–25 species of trees and shrubs widely distributed in dry areas of northeast Africa, Arabia, and India [7, 8]. Species of the genus, known as frankincense trees (olibanum), produce a resin gum traditionally harvested by making incisions on the trunks of the trees; as the resin is exposed, it darkens and hardens before being removed from the incision [9]. Among the species of the genus, only *B. serrata* Roxb. and *B. sacra* Flueck have economic importance.

The taxonomy of *Boswellia* (five species) except *B. papyrifera* (Del.) Hochst. in northern Somalia and southern Arabia has been revised. Therefore, the two extreme populations of *Boswellia spp*. vary in habitat, indumentum and shape of leaflets, number of inflorescence branches, and shape of fruits. There are also populations showing the intermediate characteristics of the extremes in Somalia and the Arabian Peninsula. Based on flower and fruit features, *B. sacra* is closely related to *B. papyrifera*. The genus has been reduced to two species, including *B. sacra* Flueck and *B. frereana* Birdwood. Based on morphological data, *B. carterii* Birdwood has been synonymized with *B. sacra* [10]. However, recent chemical analysis has shown that these are distinct species [11]. To confirm this data, further taxonomical research is required. *B. sacra* is typified as follows:

*Boswellia sacra* Flueckiger, Lehrbuch der Pharmakognosie des Pflanzenreiches [12].

Syn.: *B. carteri* Birdwood var. *subintegra* Engel., *B. bhau-dajiana* Birdwood var. *serrulata*, *B. carteri* Birdwood var. *undulate-crenata* Engel. *B. undulato-crenata* (Engl.) Engel. [10].

### 2.1. Botanical Description, Habitat, and Distribution

*B. sacra* is a tree with a distinct trunk that grows to a height of 1.5–8 m. The stems branch from the base, having pale yellowish-brown bark. The young stems can be either hairy or smooth. Resin is copious on the bark. It is milky when first exposed, then pale yellowish when dried. Leaves are densely crowded, arranged as alternates and imparipinnate with 13–19 leaflets. The inflorescence is either raceme or panicle. The flowers emerge synchronously with leaves. Pedicel length is 2–8 mm, covered with sparse hairs or smooth. Calyx is cup-shaped; the color is reddish-brown and reaches between 2 and 2.5 mm in length. Petals are white and elliptic, and the dimension is 4-5 × 2–2.5 mm. Stamens are numerous, approximately 10. The filament texture is smooth; the length is 2.5–3 mm. The anthers are oblong and white. The texture can be hairy or smooth. The length is 0.8–1.4 mm. The flowers are yellowish to orange. The pistil is furrowed, smooth, and 2.5–3 mm in length. The fruit is pyriform, reddish-brown, and has 3–4 locules. The dimension is 8–12 × 3.5–9 mm. Pyrenes are trigonous and often surrounded by a persistent wing. The dimension is 3.5–5.5 × 2-4.5 mm.

Carter first collected this species from Arabia [13]. He incorrectly named the species as *B. thulifera* and *B. serrate*. Flueckiger [14] identified it as *B. papyrifera* and later correctly identified it as a new species “*B. sacra*” in 1867 [10].

**Figure 2:**
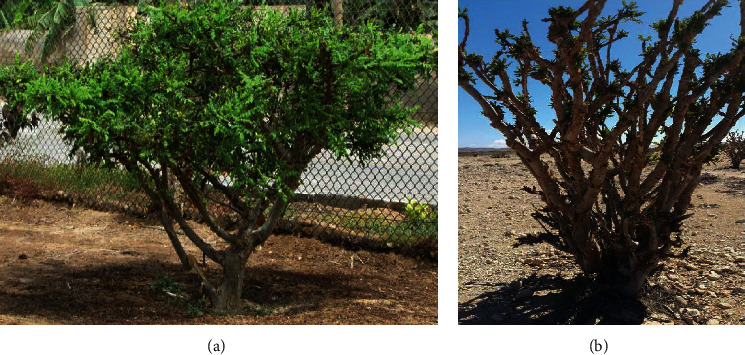
Photographs of (a) tree growing in control habitat; (b) tree growing in wild habitats given by Khan et al. [5].

*B. sacra* is mainly a saxicolous species. The cushion or disklike swelling base of the trunk adheres the plant to the rock faces. This plays an important role in stabilizing the plant, especially in very steep terrain. This species is distributed in Somalia, most parts of the Horn of Africa, and up to the Arabian Peninsula [15]. The plant growing in control and wild habitats is shown in Figure 2.

## 3. Traditional Medicine

The resin of frankincense is commonly used to treat bronchial and urinary infections. It is used as a rejuvenating medicine and treats menstrual pain, mouth problems, wounds, sores, ulcers, carbuncles, hemorrhoids, inflammation, and throat problems in some Asian countries. It is also known that frankincense oil has carminative, digestive, and diuretic properties. This gum resin's water extract, known as “Moh-Lubban,” is traditionally used to treat coughs and stomach problems [1].

In Oman, *B. sacra* gum extracts have long been used in various folk medicines for strengthening and stimulating the digestion process, dental infections, and for the treatment of colds, cough, muscle pain, fever, and asthma, as well as different types of cancer [16, 17]. In Arabian folk medicine, the gum resin is reputed to improve memory [18].

## 4. Chemical Composition

Frankincense gum resin contains 5–9% oil, a 60–70% alcohol-soluble fraction, and a 25–30% water fraction. The lipophilic part is a rich source of terpenoids, especially the medicinally important group of boswellic acids (BAs) [6, 19]. Plant resins are lipid-soluble mixtures of volatile and nonvolatile terpenoids [5].

### 4.1. Volatile Terpenoids

**Figure 3:**
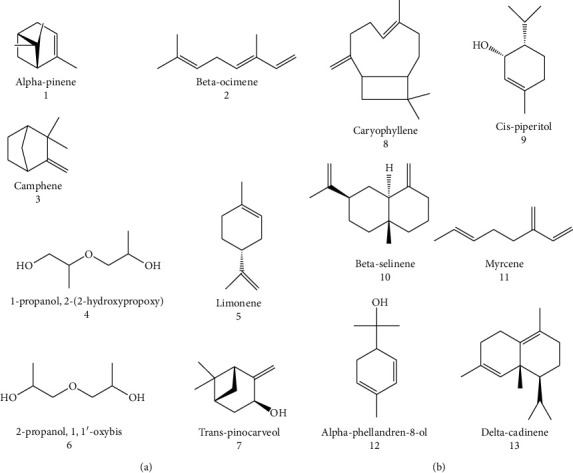
The chemical composition of the volatile oil of *B. sacra* resin.

The chemical composition of the volatile oil of *B. sacra* resin has been investigated by GC-FID, GC/MS, and headspace SPME methods that revealed *α*-pinene (38%) (1), *β*-ocimene (32.3%; 2), camphene (29.4%; 3), 1-propanol, 2-(2-hydroxypropoxy) (14.4%; 4), limonene (13.36%; 5), and 2- propanol, 1, 1′-oxybis (11.2%; 6) as the main compounds. Also, other compounds such as trans-pinocarveol (3.98%) (7), caryophyllene (3.03%; 8), cis-piperitol (2.53%; 9), *β*-selinene (2.49%; 10), myrcene (2.38%; 11), *α*-phellandren-8-ol (2.37%; 12), and delta-cadinene (2.21%; 13) have been reported in significant amounts in the essential oil from *B. sacra* (21–23) (Figure 3).

### 4.2. Sesquiterpenoids

**Figure 4:**
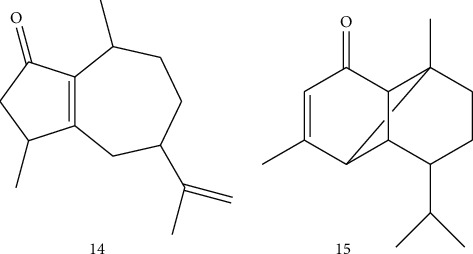
Sesquiterpenoids isolated from *B. sacra*.

Investigation of *B. sacra* led to the isolation and identification of two oxygenated sesquiterpenes, namely, rotundone (14) and mustakone (15) [24] (Figure 4). These compounds were isolated from the volatile oil of gum resin by sensory-guided fractionation [24].

### 4.3. Diterpenoids

**Figure 5:**
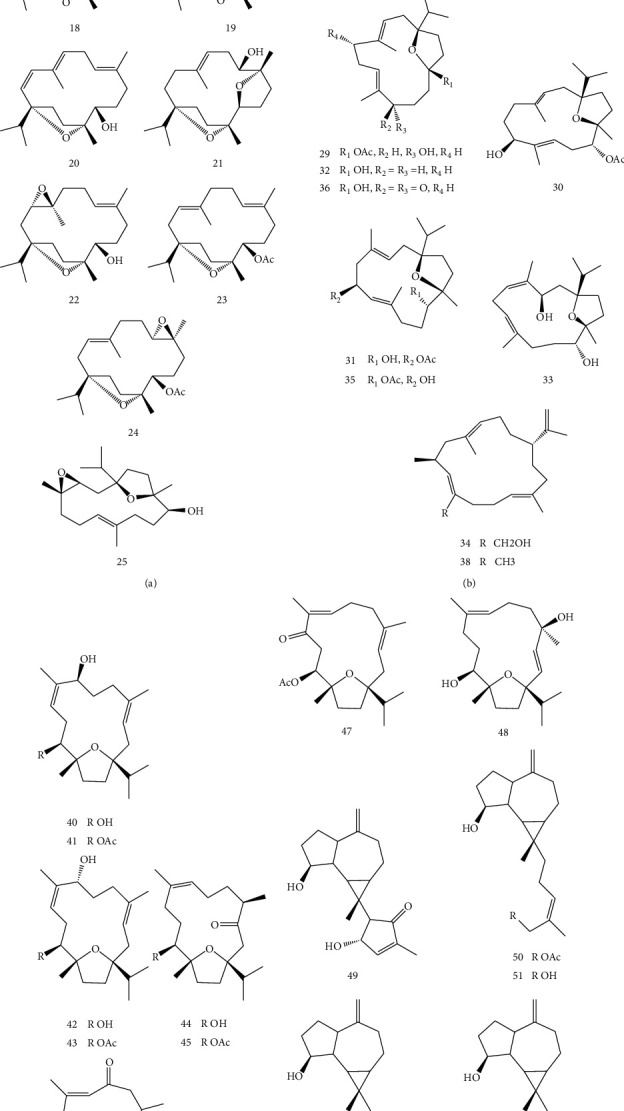
Structure of isolated diterpenoids from *B. sacra*.

Four new cembrane-type diterpenoids, including boscartins (16–19), together with five known compounds (1S, 3 R, 11S, 12 R, 7E)-1,12-epoxy-4-methylenecembr-7-ene-3,11-diol (20), isoincensole oxide (21), incensole oxide (22), incensole acetate (23), and incensole oxide acetate (24) were isolated from *B. sacra* gum resin by Wang et al. In addition, hepatoprotective properties of isolated compounds were studied against HepG2 cells that had been damaged by paracetamol compared to bicyclol (as a positive control). Incensole acetate had a potent hepatoprotective effect at 10 *μ*M. In contrast, boscartin M, isoincensole oxide, incensole oxide, and incensole oxide acetate had a mild hepatoprotective effect at 10 *μ*M [25]. In the other report by Wang et al., the investigation of *B. sacra* led to the isolation of ten new cembrane-type diterpenes, including boscartins AL-AU (25–34) and five known analogs (35–39). Moreover, biological evaluations revealed that compounds 27, 29, 36, and 37 displayed hepatoprotective activities against paracetamol-induced HepG2 cell damage at 10 *μ*M. Some compounds exhibited moderate neuroprotective activities in two different models [26]. Zhang et al. isolated eight diterpenoids (40–48), namely, sacraoxides A–G from the gum resin of *B. sacra* and found that sacraoxides E and F had inhibitory activities on nitric oxide (NO) production induced by lipopolysaccharide in RAW264.7 cells with IC_50_ values of 24.9 ± 1.7 and 36.4 ± 2.9 *μ*M [27]. In another study, five diterpenoids (49–53) including two new prenylaromadendrane-type diterpenoids, and three known analogues, were isolated from the ethanol extract of the gum resin of B. sacra Flueck by Wang et al.. All compounds exhibited notable cytotoxicity against human malignant glioma (U87-MG) cell line against 5-fluorouracil as a positive control [28].  Figure 5 shows the structures of isolated diterpenoids from *B. sacra*.

### 4.4. Triterpenoids

**Figure 6:**
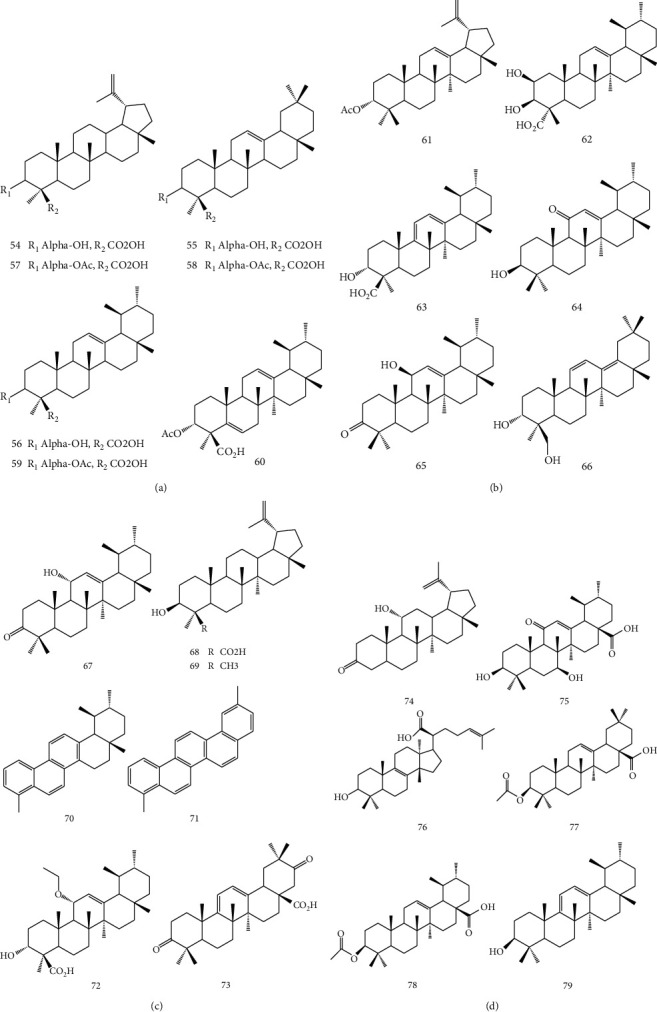
Structure of isolated triterpenoids from *B. sacra*.

*B. sacra* could be chemically characterized by the occurrence of triterpenoid compounds such as lupeolic acid, *α*- and *β*-boswellic acids (54, 55, and 56), and their respective O-acetyl derivatives (57, 58, and 59) [28]. Ali et al. isolated two new O-acetyl derivatives of pentacyclic triterpenic acids, 3*α*-acetoxyurs-5:12-dien-24-oic acid (60) and 3-acetoxylup-12 : 20-dien-24-oic acid (61) [29] from Omani frankincense of *B. sacra* along with four known compounds: commic acid-D (62), 9,11-dehydro-boswellic acid (63), 3- hydroxy-11-oxours-12-ene (64), and 11-hydroxy-3-oxours-12-ene (65) [30]. One ursane-type (66), one oleanane-type (67), namely, olean-11,13(18)-dien-3*β*,24-diol and 3-oxo-11a-hydroxy-urs-12-ene, respectively, as well as two lupane-type triterpenoids, lupeolic acid (68) and lupeol (69), have been reported by Al-Harrasi et al. from the resin of *B. sacra* [17]. The frankincense resin of *B. sacra* was pyrolyzed, and the smoke was trapped into water using a self-developed assembly. Two compounds, namely, 1,2,4a,9-tetramethyl-1,2,3,4,4a,5,6,14b-octahydropicene (70) and 2,9-dimethylpicene (71) were isolated from an n-hexane extract of the smoke-saturated water. Compounds 70 and 71 were evaluated for their antiproliferative activity against MDA-MB-231 breast cancer cells, and it was observed that these pyrolysate products were able to inhibit cancer cell growth [31]. A new ursane-type triterpene, namely, nizwanone (72) was reported from Omani frankincense *B. sacra* Flueck along with two known compounds papyriogenin B (73) and rigidenol (74) by Al-Harrasi et al. [32]. 11-keto-ursolic acid (75), 3*α*-hydroxy-8, 24-dien-tirucallic acid (76), 3-O-acetyl-oleanolic acid (77), and 3-O-acetyl-ursulic acid (78) were reported as triterpenoid compounds from a methanolic extract [33]. Similarly, ten more known compounds were isolated from the resin of *B. sacra* including one triterpene (79) [34]. Structures of isolated triterpenoids from *B. sacra* are shown in Figure 6.

### 4.5. Boswellic Acids and Derivatives

**Figure 7:**
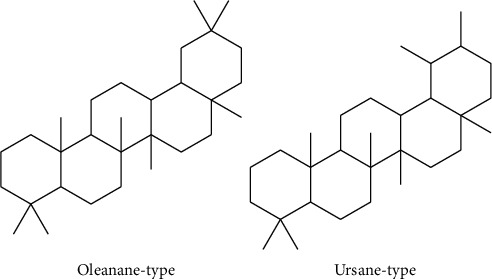
Basic structure of triterpenoids in BAs.

The primary active components from the extract of *B. sacra* are boswellic acids (BAs). As shown in Figure 7, BAs are a group of oleanane or ursane pentacyclic triterpenoids with carboxylic acid at C- 4 and are divided into two groups, *β*-BAs (ursane-type) and *α*-BAs (oleanane-type). Their potency against inflammation, arthritis, ulcerative colitis, chronic colitis, asthma, and hepatitis is well documented. They also have exhibited antimicrobial, antidiabetic, antiviral, and antipruritic activity. One of the important uses of BAs in medicine is as an anti-inflammatory. Boswellic acids suppress leukotriene biosynthesis in neutrophilic granulocytes by nonredox, noncompetitive inhibition of 5-lipoxygenase [32, 35–37]. The pharmacological effects of *B. sacra* extract are commonly explained by the presence of boswellic acids [36]. Also, the anticancer activity of BAs is remarkable. In this regard, they have activity against cancers including bladder, brain, cervical, colon, colorectal, liver, leukemia, lung, melanoma, meningioma, multiple myeloma, neuroblastoma, ovarian, pancreatic, and prostate [38].

**Figure 8:**
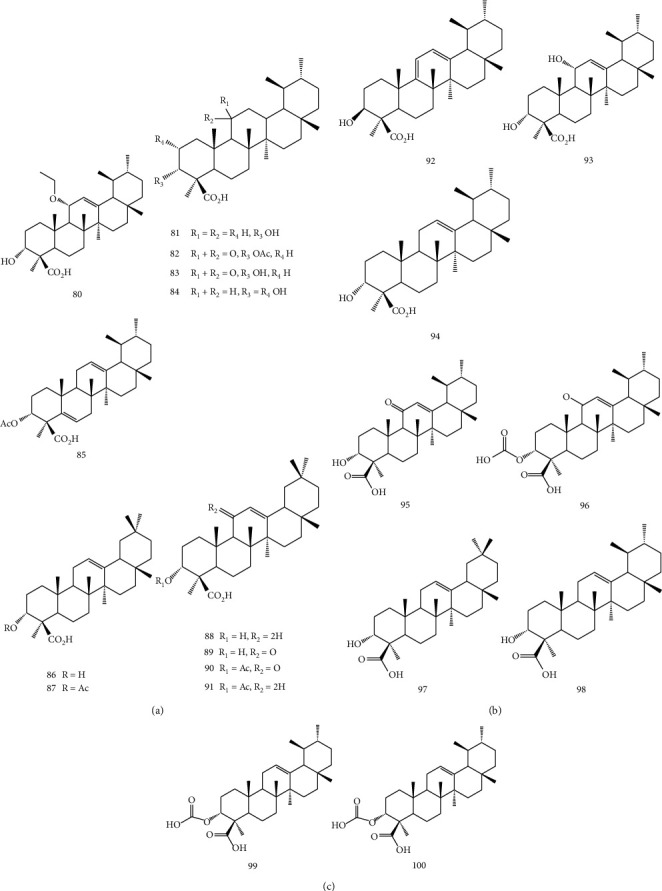
Structure of isolated boswellic acids and derivatives from *B. sacra*.

A new boswellic acid derivative, 11*α*-ethoxy-*β*-boswellic acid (EBA; 80), was isolated from Omani frankincense *B. sacra* Flueck by Al-Harrasi et al. [32]. They also reported the isolation of five boswellic acid derivatives (81–85) from the resin of *B. sacra* [17]. Similarly, ten more known compounds were isolated from the resin of *B. sacra* such as nine boswellic acids (86–91 and 92–94). Compounds 87 and 89–91 were found to be significantly active against *α*-glucosidase with an IC_50_ value ranging from 15.0 ± 0.84 to 80.3 ± 2.33 *μ*M, while 92 exhibited moderate activity with an IC_50_ of 799.9 ± 4.98 *μ*M [34]. 11-keto-*β*-boswellic acid (95), 3-O-acetyl-11-keto-*β*-boswellic acid (96), *α*-boswellic acid (97), *β*-boswellic acid (98), 3-O-acetyl- *α*-boswellic acid (99), and 3-O-acetyl-*β*-boswellic acid (100) were isolated as boswellic acid derivatives from a methanolic extract [33]. Structures of the isolated boswellic acids and their derivatives from *B. sacra* are shown in Figure 8.

## 5. Standardization

The used part of the plant is an oleo gum resin that is easily found in herbal stores. For standardization of the oleo gum resin, boswellic acids and their derivatives have been accounted as standard markers [1].

## 6. *B. sacra* Products

**Figure 9:**
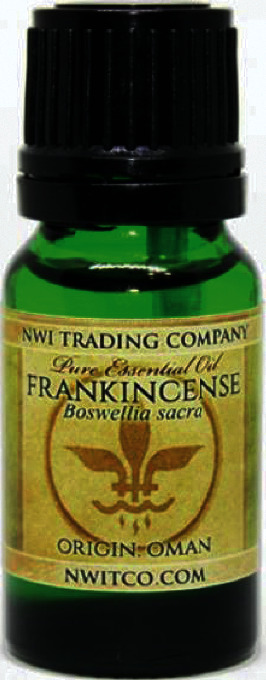
Frankincense *B. sacra* essential oil (https://www.amazon.com).

There are *B. sacra* essential oils as liquid form, for example in 10 ml bottles (Figure 9).

## 7. Bioactivities and Professional Pharmaceutical Applications

### 7.1. Anticancer Activity

The essential oil of *B. sacra* has been found to induce breast cancer cell-specific cytotoxicity. *B. sacra* essential oil can suppress tumor aggressiveness in drug-resistant and cultured metastasized human breast cancer cells. *B. sacra* showed proapoptotic, antiproliferative, and anti-invasive properties in human breast cancer cell lines [39]. Essential oil from *B. sacra* suppresses viability and induces apoptosis in human pancreatic cancer cell lines. Frankincense essential oil activates the caspase-dependent apoptotic pathway, Akt and Erk1/2 signaling molecules and suppresses the levels of cyclin D1 (an important cell cycle regulator) expression in human pancreatic cancer cells. Essential oil of *B. sacra* gum resin has been suggested as a useful alternative therapeutic agent for patients with pancreatic adenocarcinoma, the major type of aggressive pancreatic cancer [40]. Besides, another study proposed that the essential oil was potent against colon cancer cells such as CD133+ and CD133-Colo-320 cells, and it was also considered that essential oil obtained from B. sacra led to a reduction of *β*-catenin signaling molecules which have a vital role in cancer cell proliferation [41]. In a case report study, oral administration of *B. sacra* gum resin was reported to be effective in urothelial cell carcinoma [42].

### 7.2. Analgesic Effects

The analgesic effects of crude extracts and fractions of Omani frankincense obtained from *B. sacra* were studied in two mouse models of pain. Administration of the extract, essential oils, and subfractions from the resin of *B. sacra* in acetic acid-induced writhing and formalin tests demonstrated the antinociceptive properties of *B. sacra* as a traditional medicinal plant [16].

### 7.3. Hepatoprotective Effects

Biological evaluations of cembrane-type diterpenes isolated from *B. sacra* showed that some of these compounds exhibited obvious hepatoprotective effects against paracetamol-induced HepG2 cell damage [26]. Administration of the water extract of oleo-gum-resin of *B. sacra* in rats demonstrated that this plant possesses hepatoprotective activity against carbon tetrachloride-induced acute and chronic hepatic damage [43].

### 7.4. Antimicrobial Effects

Essential oils from *B. sacra* have been traditionally used to treat microbial and fungal infections. *In vitro* study of monoterpenoids of *B. sacra* essential oil showed antimicrobial activity against *Staphylococcus aureus*, *Pseudomonas aeruginosa*, and *Propionibacterium acnes*. Frankincense essential oils also exhibited a significant antifungal effect against *Candida albicans* and *Malassezia furfur* [2].

The inhibitory effect of different concentrations of *B. sacra* resin, leaf extract, and essential oil has been evaluated on the growth and production of aflatoxins by *Aspergillus flavus* and *Aspergillus parasiticus*. This study revealed that the resin powder and essential oil of *B. sacra* markedly reduce aflatoxin production. Therefore, the resin powder and essential oil of *B. sacra* can be recommended as safe natural food preservatives to increase the shelf life of food and feed products with reference to their antimicrobial and aflatoxin inhibitory activities [44]. In another study, it was found that *B. sacra* oleoresin extract has a promising antibacterial and antibiofilm activity against *Porphyromonas gingivalis* [45]. Moreover, the potential of *B. sacra* extract was evaluated against a number of human gastrointestinal bacterial pathogens and autoimmune disease-stimulating bacteria in combination with conventional antibiotics. In this regard, the obtained results confirmed that the combination of conventional antibiotics with *B. sacra* extracts exhibited extremely higher activity than that of the individual components alone [46]. Recently, a research study revealed that B. sacra essential oil exhibited promising antifungal activity against some fungi species which lead to strawberry rot such as *Botrytis cinerea*, *Aspergillus niger*, and *Rhizopus stolonifera* [47].

### 7.5. Antioxidant Effects

It has been shown that the essential oil of *B. sacra* gum resin has a strong antioxidant effect in the DPPH radical scavenging method. However, its antioxidant activity is lower as compared to the antioxidant property of ascorbic acid [48]. The effect of *B. sacra* oleo gum resin extract has been studied against testicular toxicity in rats. The extract of this plant decreased the gene expression of HSP70 (heat shock protein-70), GSTPi (glutathione-s-transferase-Pi), and IGFBP3 (insulin-like growth factor binding protein-3) in the testes. The result of this study suggests that the antioxidant effect of *B. sacra* may protect the testes against several toxicants through the inhibition of free radicals [1]. Moreover, recent evidence indicated that incorporation of *B. sacra* essential oil into nanoparticles enhanced the efficacy of some pharmacological properties such as antioxidant activity [49].

### 7.6. Anti-Alzheimer Effect

The genus *Boswellia* has been suggested to cure or prevent neurodegenerative disorders through anti-inflammatory, antioxidative, antiamyloidogenic, and antiapoptotic effects [50]. Evaluating the effect of essential oil obtained from resins of *B. sacra* showed that frankincense essential oil can significantly inhibit the acetylcholinesterase enzyme (AChE). Inhibition of AChE leads to increased acetylcholine levels in the brain and improves memory in Alzheimer's disease patients. Therefore, *B. sacra* as a medicinal plant may protect against memory loss from Alzheimer's disease [51].

### 7.7. Anti-Inflammatory Effect

The anti-inflammatory effects of essential oil from *B. sacra* have been studied on an ovalbumin-induced allergic asthma mouse model. The results of this evaluation showed that inhalation of *B. sacra* essential oil has a potential therapeutic effect in allergic airway inflammation through increasing Th1 cytokine (IFN-*γ*) and decreasing Th2 cytokines (IL-4, IL-5, and IL-13) levels [52].

### 7.8. Antiseizure Effect

More recently, Wolfender and coworkers reported a comprehensive study on the antiseizure activity of resin of *B. sacra*. Results of this study demonstrated that among all isolated terpenoids, *β*-boswellic acid which belongs to triterpenoid derivatives was the most active and resulted in a 90% reduction of pentylenetetrazole (PTZ)-induced seizures at 100 *μ*g/mL [53]. The pharmacological activity of *B. sacra* gum resin and its phytochemicals is summarized in Table 1.

## 8. Future Perspectives

It is strongly believed that detailed information on the phytochemical and biological activities of *B. sacra*, as presented in this review, provides certain evidence for the use of this plant in different medicines and future pharmaceutical studies. It seems that the oleo gum resin of *B. sacra* is an important resource in the appearance of new drugs and herbal medicine formulations. However, more determining the pharmacological activity of *B. sacra*, especially in future clinical studies, is suggested. It is better that should have been a correlation between traditional uses of *B. sacra* and new studies. Further studies are needed to isolate the active compounds for the observed pharmacological activities. Also, the herbal medicine formulation should be prepared and standardized on the basis of the active compounds. Further studies should be focused on the mechanisms behind the anti-inflammatory and memory improvement activities and those biological activities that have been reported traditionally.

## 9. Conclusion

This present work was designed to show that *B. sacra* is a valuable medicinal plant and an interesting subject to researchers. Many studies have indicated that *B. sacra* is a rich source of terpenoid compounds, especially triterpenoids, responsible for a wide range of biological activities. Although different studies have investigated these, there is no data on the clinical effectiveness of *B. sacra*. The findings of this review study on *B. sacra* suggested that clinical trial studies should be undertaken to explore the anti-inflammatory activity and memory improvement effects. *B. sacra* has a long and brilliant history in traditional medicine, and that is why clinical trial studies are strongly recommended for its drug development.

## Figures and Tables

**Table 1:** The pharmacological profiles of *B. sacra* gum resin and its phytochemicals.

Activity	Extract/compounds	Dose and treatment period	Model	Results	Mechanism	Reference
Anticancer	Essential oil, 11-keto-b-boswellic acid	100 *μ*l, treat, 24 h	T47D, MCF7, MDA-MB-231, MCF10-2A	Reduced cell viability, elevated cell death, suppressed cellular network formation, disrupted spheroid development of breast cancer cells, caused the breakdown of multicellular tumor spheroids (T47D), no cytotoxity on MCF10-2A, inhibited caspases in the MDA-MB-231, anti-invasive	(+) cleaved caspase-3(−) caspase-8 p43/p41, caspase-9 p37/p35, pro-caspase-3, Akt, PARP, ERK1/2(Thr202/Tyr204), cdk4, cyclin D1	[32, 35, 39]
	Essential oil, 11-keto-b-boswellic acid	100 *μ*l, treat, 24 h	MIA PaCa-2, Panc-28, BxPC-3, DANG			[35, 40]
	Essential oil	30 *μ*l, SQI, 3 days	Heterotopic xenograft mouse	Suppressed viability, reduced cell growth, antiproliferative, antiapoptotic, induced cytotoxicity, reduced tumor volume, anti-tumor	(+) Caspase-3 activation, apoptosis(−) cyclin D1, cdk4, caspase-8 p43/p41, caspase-9p37/p35, procaspase-3, PARP, Akt ser (473), ERK1/2(Thr202/Tyr204), proliferating	[42]
		3 mL daily, oral, 25 months	A 52-year-old male with urothelial cell carcinoma		(+) No(−) AST, ALT, bilirubin, creatinine, BUN	
	Methanolic extract, Boswellic acids	6.25–100 *μ*g/ml, incubated, 24 and 48 h	Human pancreatic (PANC1), colon (HCT116), lung adenocarcinoma (MOR), breast cancer MCF7 and MDA-MB-231, the human prostate cancer LNCaP, and SerBob cell lines	Inhibited tumor growth, anticancer, improved kidney and liver function, decreased tumor volume	(+)-(−)-	[55]
				Anti-tumor cytotoxicity, inhibited cell viability		
Anti-inflammatory	Essential oil	0.3%, inhalation, 8 weeks	Ovalbumin-induced BALB/c mice allergic asthma	Reduced eosinophils, decreased goblet cell hyperplasia, inhibited hyperresponsiveness, anti-inflammatory, immunity	(+) Th1, IFN-*γ*, (−) Th2, IL-4, IL-5, IL-13, CD4^+^, CD3^+^/CCR3^+^, B220^+^/CD23^+^	[51]
Analgesic	Methanolic extract, essential oils, subfractions	300 mg/kg, orally	Acetic acid-induced writhes and formalin-induced pain in mice	Analgesic, inhibited writhes, inhibited licking, and biting response	(+) No(−) No	[32]
Antimicrobial	Essential oil	0.1 ml, treat, 24 h	*Staphylococcus aureus* (ATCC 25923; ATCC 6538), *Pseudomonas aeruginosa* (ATCC 15442; ATCC 9027), *Candida albicans* (ATCC 10231), *Malassezia furfur* (ATCC 14521)	Antifungal, antimicrobial	(+) No(−) No	[2]
	Resin essential oil	2.5, 5, 7.5, 10 g/100 ml1, 2, 3, 4 ml/100 ml, incubated, 15 days	*Aspergillus flavus* (SQU21), *Aspergillus parasiticus* (CBS921.7)	Inhibited microbial and aflatoxins, enhanced fungal growth, inhibited aflatoxin biosynthesis and secretion pathway	(+) Mycelial dry weights(−) Inhibited fungal growth and aflatoxins production	[4]
Anti-Alzheimer	Essential oil, (+) pinene	0.5 mg/ml, incubated, 15 min	AChE, Jack bean urease	Anti-Alzheimer's disease, protected stomach ulcers	(+) No(−) AChE, urease enzyme	[50]
	Diterpenoids of ethanolic extract	10 *μ*M, incubated,1 h	Glutamate-induced toxicity rat cortical neurons, human neuroblastoma SK-N-SH cellsAChE model	Neuroprotection	(+) No(−) No	[26]
	Boswellic acids, ethyl acetate fraction	0.75 mM, 0.23, 0.46,0.93 mg/ml, incubated, 20 min		Anti-Alzheimer's disease	(+) No(−) AChE	[18]
Hepatoprotective	Diterpenoids of ethanolic extract	10 *μ*M, incubated,1 h	Paracetamol-induced HepG2 cells	Hepatoprotective, inhibited damage	(+) No(−) No	[25, 26]
	Methanolic extract	250, 500 and 1000 mg/kg, oraly, 28-day	Safety and toxicological studies on rat	Reduced mean cellular hemoglobin (MCH), induced hypochromic normocytic anemia, may not be safe to use	(+)-(−)-	[55]
Wound healing	Methanolic extracts	10–20 *μ*g/ml, incubated, 24 h	H_2_O_2_- induced injury on adult human dermal fibroblasts	Improved proliferation, migration, and wound healing process	(+) pERK/ERK(−) ROS	[56]

*Note.* (+): increased or activated; (−): decreased or inhibited.
